# Preoperative Localization in Colonic Surgery (PLoCoS Study): a multicentric experience on behalf of the Italian Society of Colorectal Surgery (SICCR)

**DOI:** 10.1007/s13304-021-01180-7

**Published:** 2021-10-05

**Authors:** Michele Manigrasso, Marco Milone, Mario Musella, Pietro Venetucci, Francesco Maione, Ugo Elmore, Gaetano Gallo, Roberto Perinotti, Giovanni Domenico De Palma, PLoCoS Study Group, Giovanni Sarnelli, Nicola Gennarelli, Sara Vertaldi, Giuseppe Sammarco, Giuseppina Vescio, Vincenzo Tiesi, Francesco Pata, Donato Francesco Altomare, Arcangelo Picciariello, Vincenzo Papagni, Leonardo Vincenti, Massimiliano Mistrangelo, Edoardo Forcignanò, Antonio Salzano, Andrea Bondurri, Anna Maffioli, Francesco Colombo, Andrea Lauretta, Giuseppe Sica, Michela Campanelli, Marco Stella, Paolo Boati, Francesco Ferrara, Francesco Selvaggi, Gianluca Pellino, Francesco Maria Romano, Lucio Selvaggi, Yves Panis, Alice Frontali, Giovanni Spiezio, Antonino Spinelli, Francesca Di Candido, Annalisa Maroli, Claudio Coco, Gianluca Rizzo, Elisabetta Moggia, Gaetano Luglio, Gianluca Pagano, Francesca Paola Tropeano, Roberto Peltrini, Federico Marchesi, Gabriele Luciano Petracca, Giorgio Dalmonte, Marina Valente, Antonio Giuliani, Harmony Impellizzeri, Enrico Marrano, Gianluigi Moretto, Cristina Folliero, Antonio Langone, Giuseppe Caristo, Giorgio Maria Paolo Graziano, Angelo Amico, Antonio Di Cataldo, Pietro Maida, Ester Marra, Roberta Abete, Antonio Castaldi, Alessio Palumbo, Fabrizio Foroni, Carmine Antropoli, Paola De Nardi, Roberto Quattromani, Riccardo Rosati

**Affiliations:** 1 Department of Advanced Biomedical Sciences, “Federico II” University of Naples, Via Sergio Pansini 5, 80131 Naples, Italy; 2 Department of Clinical Medicine and Surgery, “Federico II” University of Naples, Via Pansini 5, 80131 Naples, Italy; 3 Division of Gastrointestinal Surgery, San Raffaele Scientific Institute, 60 Via Olgettina, 20132 Milan, Italy; 4 Operative Unit of General Surgery, Department of Medical and Surgical Sciences, University of Catanzaro, Catanzaro, Italy; 5 Colorectal Surgical Unit, Department of Surgery, Infermi Hospital, Biella, Italy

**Keywords:** Colorectal, Colonoscopy, CT scan, Localization, Colon cancer, Surgery

## Abstract

The aim of this prospective multicentric study was to compare the accurate colonic lesion localization ratio between CT and colonoscopy in comparison with surgery. All consecutive patients from 1st January to 31st December 2019 with a histologically confirmed diagnosis of dysplastic adenoma or adenocarcinoma with planned elective, curative colonic resection who underwent both colonoscopy and CT scans were included. Each patient underwent conventional colonoscopy and CT to stage the tumour, and the localization results of each procedure were registered. CT and colonoscopic localization were compared with surgical localization, adopted as the reference. Our analysis included 745 patients from 23 centres. After comparing the accuracy of colonoscopy and CT (for visible lesions) in localizing colonic lesions, no significant differences were found between the two preoperative tools (510/661 vs 499/661 correctly localized lesions, *p* = 0.518). Furthermore, after analysing only the patients who underwent complete colonoscopy and had a visible lesion on CT, no significant difference was observed between conventional colonoscopy and CT (331/427 vs 340/427, *p* = 0.505). Considering the intraoperative localization results as a reference, a comparison between colonoscopy and CT showed that colonoscopy significantly failed to correctly locate the lesions localized in the descending colon (17/32 vs 26/32, *p* = 0.031). We did not identify an advantage in using CT to localize colonic tumours. In this setting, colonoscopy should be considered the reference to properly localize lesions; however, to better identify lesions in the descending colon, CT could be considered a valuable tool to improve the accuracy of lesion localization.

## Introduction

Although colonoscopy is currently considered the method of choice to detect colorectal cancer, little is known about its accuracy in tumour localization [1, 2].

In fact, colonoscope orientation throughout the colonic segments is complicated by the absence of specific anatomic landmarks between the anal verge and the ileocecal valve, resulting in inaccurate lesion localization in 11–21% of cases [3–6].

Inaccurate localization plays a critical role during planned surgical procedures, especially laparoscopic and robotic surgery, in which trocar positioning or arms docking are fundamental to performing the correct surgical intervention [7–9].

In this setting, computed tomography (CT) could be considered another approach for correctly localizing colonic lesions, as it is able to locate the major colonic anatomical landmarks, caecum and colonic flexures [10].

The aim of this prospective multicentric study was to compare CT and colonoscopy in terms of their accuracy in localizing colonic lesions.

## Materials and methods

The study was approved by our institutional review board, and informed consent was obtained from all subjects before enrolment. A 1-year prospective observational study enrolling all consecutive patients in tertiary referral colorectal centres from 1st January to 31st December 2019 with a histologically confirmed diagnosis of colon dysplastic lesion or cancer was performed. The study findings have been reported in compliance with the STROBE checklist [11].

All patients with a histologically confirmed diagnosis of dysplastic adenoma or adenocarcinoma with planned elective, curative colonic resection who underwent both colonoscopy and CT were included. The exclusion criteria were as follows: rectal cancer detected during preoperative staging; inability to perform preoperative colonoscopy or CT; emergency surgery; curative endoscopic treatment; and death before surgery.

Each patient underwent conventional colonoscopy and CT to stage the tumour, and the localization results of each procedure were registered. CT and colonoscopic localization were compared with surgical localization, which was adopted as the reference standard.

Colonoscopic localization was performed by an expert endoscopist (at least 500 colonoscopies per year). Furthermore, an endoscopist indicated the colon tract affected by the lesion, choosing among eight segments as shown below.

CT exams were performed by an expert radiologist (at least 300 CT scans per year) with multislice devices and at least 64 slices.

Briefly, first, a “scout exam” of the abdomen and the pelvis was performed, followed by a multiphasic CT study. The latter was first performed without contrast medium and then during the venous phase after an injection of iodate contrast medium. In both phases, with and without contrast, the whole abdomen and pelvis were checked to be sure that the colon was completely included. In selected cases, integrative scans were performed, especially if patients already underwent colonic surgery. The radiologist indicated the colon tract in which the lesion was present, choosing among the colonic segments as shown below.

The endoscopists and radiologists were blinded to the lesion localization results.

Finally, the lesion location was intraoperatively identified by the surgeon. The type of intervention (open surgery or minimally invasive approach) was chosen by each surgeon according to his/her preferences.

**Fig. 1:**
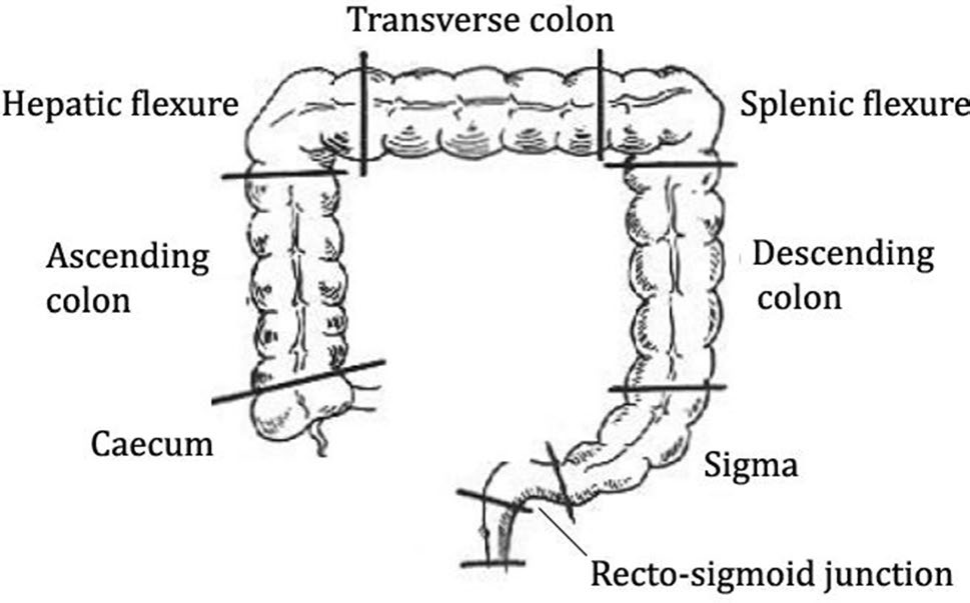
Division of the colon in eight segments

In each procedure, the colon was divided into 8 parts (Fig. 1):

- *Rectosigmoid junction*: between the last Houston valve and the first tract of the sigmoid segment;
- *Sigmoid*: tortuous segment between 40 and 15 cm from the anus (last Houston valve);
- *Descending colon*: straight segment between 10 cm from the splenic flexure and 40 cm from the anus;
- *Splenic flexure*: tract between 10 cm before and after the splenic curve of the colon;
- *Transverse colon*: tract between 10 cm after the splenic flexure and 10 cm before the hepatic flexure;
- *Hepatic flexure*: tract between 10 cm before and after the hepatic curve of the colon;
- *Ascending colon*: tract between 10 cm proximal from the hepatic curve and ileocecal valve;
- *Caecum*: tract limited by the ileocecal valve and the bottom of the caecum with the appendix orifice.

### Data analysis and outcomes assessment

For the included patients, data on sex, age, body mass index (BMI), American Society of Anaesthesiologists (ASA) score and previous colonic surgery were recorded.

During colonoscopy, in addition to lesion localization, data about the colonoscopy procedure and the presence of an obstructing mass were recorded. During the CT scans, data about the visibility of the lesion were recorded. Intraoperative data included the type of intervention (open, laparoscopic, or robotic approach), the need to modify the surgical approach because of an incorrect lesion localization and the type of the modified approach.

Finally, the tumour characteristics included T stage and the maximum diameter of the lesion, expressed in centimetres (cm).

The primary outcome was the accurate lesion localization ratio of conventional colonoscopy and computed tomography in localizing colonic lesions, in comparison with surgery.

Accurate localization was defined as the ratio of colonoscopic or imaging localization with intraoperative localization (considered the true value) and was expressed in percentage.

The secondary outcome was patient and disease characteristics (age, sex, BMI, previous colonic surgery, T stage and tumour size) that influenced correct localization of the lesion.

### Statistical analysis

Statistical analyses were performed with SPSS 26.0 (SPSS Inc., IBM, Chicago, IL, USA).

Continuous variables are expressed as the mean ± standard deviation (SD); categorical variables are expressed as percentages (%). Continuous variables were compared by the Mann–Whitney *U* test and *t*-test, and categorical variables were compared by the Chi-square test. When the minimum expected value was less than five, we adopted Fisher’s exact test. A *p* value < 0.05 was defined as statistically significant.

The agreement between the two diagnostic methods was calculated using the weighted Cohen κ statistics. The κ values were considered as follows: 0–0.20, slight agreement; 0.21–0.40, fair agreement; 0.41–0.60, moderate agreement; 0.61–0.80, substantial agreement; and 0.81–1 almost perfect agreement.

A multivariate analysis (stepwise method) was adopted to identify tumour and patient factors independently associated with incorrect lesion localization by each preoperative procedure, expressed by the odds ratio (OR) and 95% confidence interval (95% CI).

## Results

Our analysis included 745 patients from 23 centres (22 across Italy and one in France).

Of these patients, one was excluded because he died before CT, seven patients were excluded because of the rectal location of the neoplasia during preoperative staging, two were excluded because of the inability to perform colonoscopy (no compliance with bowel preparation), and six patients were excluded because the lesion was endoscopically treated. Thus, the final analysis involved 729 patients.

### Demographic and pathological data

Of the 729 included patients, 411 (56.4%) were male, the mean age was 70 ± 11.21 years, the mean ASA score was 2.36 ± 0.64, and the mean BMI was 25.84 ± 4.3 kg/m^2^; 22 patients (3%) underwent previous colonic resection. Regarding the pathological data, the lesions were dysplastic adenoma or adenocarcinoma in situ in 4.4% of cases, stage T1 in 8.8%, stage T2 in 15.8%, stage T3 in 50.8%, stage T4 in 15.9%, and not reported in 4.4%.

The maximum diameter of the lesion (dmax) was reported in 677 cases, with a mean dmax of 4.4 ± 2.16 cm. The demographic and pathological data are shown in Table 1.

**Table 1:** Demographic characteristics of the included patients and pathologic data of the lesions

Characteristics	No of patients (%)
Patients	729
Male	441 (56.4)
Female	318 (43.6)
Age (years)	70 ± 11.21
BMI (kg/m^2^)	25.84 ± 4.3
ASA score	2.36 ± 0.64
I	50 (6.9)
II	364 (49.9)
III	277 (38)
IV	15 (2.1)
Not reported	23 (3.2)
Previous colonic resection	22 (3)
T stage	
Dysplastic adenoma/ T in situ	32 (4.4)
T1	64 (8.8)
T2	115 (15.8)
T3	370 (50.8)
T4	116 (15.9)
Not reported	32 (4.4)
Lesion dmax	4.4 ± 2.16 cm

*BMI* body mass index, *dmax* maximum diameter, *cm* centimetres

### Lesion localization by colonoscopy

Colonoscopy was completed in 488 cases (66.9%). The reasons for incomplete colonoscopy were tumoral stenosis (88.8%), inadequate colon cleansing (2.5%), lack of patient compliance (0.8%), intraoperative bleeding (0.4%), and unreported reasons (7.5%).

Colonoscopy localized the lesions throughout the eight colonic segments, with most lesions located in the sigmoid (26.6%), ascending colon (21.1%) and caecum (15.1%). Comparing intraoperative and colonoscopic localization, there were small differences in all the colonic segments, but the differences were not significant.

Correct localizations were obtained in 544 cases (74.6%). The results of lesion localization by colonoscopy are summarized in Table 2.

**Table 2:** Colonoscopy data, lesion localization and comparison with intraoperative localization

Characteristics	Colonoscopy *n* = 729 (%)	Surgery *n* = 729 (%)	*p* value
Localization			
Caecum	110 (15.1)	124 (17)	0.354
Ascending colon	154 (21.1)	157 (21.5)	0.896
Hepatic flexure	67 (9.2)	49 (6.7)	0.858
Transverse colon	62 (8.5)	70 (9.6)	0.523
Splenic flexure	43 (5.9)	54 (7.4)	0.293
Descending colon	57 (7.8)	41 (5.6)	0.176
Sigma	194 (26.6)	184 (25.2)	0.591
Recto-sigmoid junction	42 (5.8)	49 (6.7)	0.516
Overall accuracy	544 (74.6)		
Incomplete colonoscopy	241 (33.1)		
Reason for incomplete colonoscopy			
Tumoral stenosis	214 (88.8)		
Inadequate colon cleansing	6 (2.5)		
No patient compliance	2 (0.8)		
Intraluminal bleeding	1 (0.4)		
Not reported	16 (7.5)		

### Lesion localization by CT

Preoperative CT visualized a colonic lesion in 90.7% of cases (661/729). Correct localization was reported in 70.1% of cases (519/729). However, when only the 661 cases in which the lesion was visualized was considered, the accuracy increased to 77.2% (510/661).

Of the 185 lesions erroneously localized by colonoscopy, 74 lesions (40%) were accurately localized by CT.

Thus, combining the correct CT localization with the incorrect colonoscopic localization, the combined accuracy reached 84.8%, localizing 618 of 729 lesions.

Similarly, on CT scans, most lesions were localized in the sigmoid (25.9%), ascending colon (20.9%) and caecum (17.7%).

In the comparison of imaging and intraoperative data, small nonsignificant differences were recorded in each colonic segment. The data on lesion localization by CT are reported in Table 3.

**Table 3:** CT scan data, lesion localization and comparison with intraoperative localization

Characteristics	CT scan (%)	Surgery (%)	*p* value
Number of detected lesions	661 (90.7)		
Localization			
Caecum	117 (17.7)	118 (17.8)	1.000*
Ascending colon	138 (20.9)	144 (21.8)	0.687*
Hepatic flexure	59 (8.9)	42 (6.3)	0.151*
Transverse colon	54 (8.1)	65 (9.9)	0.337*
Splenic flexure	42 (6.4)	52 (7.9)	0.335*
Descending colon	58 (8.8)	32 (4.8)	0.083*
Sigma	171 (25.9)	161 (24.4)	0.568*
Recto-sigmoid junction	32 (4.8)	47 (7.1)	0.104*
Overall accuracy	510 (70.1)		
Accuracy on detected lesions	510/661 (77.2)		

*Analyses are performed on 661 patients (lesions seen at CT)

### Comparison of colonoscopy and computed tomography

When comparing the accurate lesion localization ratio of colonoscopy and CT (for visible lesions), no significant differences were found between the two preoperative tools (510/661 vs 499/661 correctly localized lesions, *p* = 0.518).

Furthermore, analysing only the patients who underwent complete colonoscopy with a lesion visible on CT, the comparison of colonoscopy and computed tomography showed no significant difference in correct lesions localization (331/427 vs 340/427 correctly localized lesions, *p* = 0.505).

Considering the intraoperative localization as a reference, a comparison between colonoscopy and CT showed that colonoscopy significantly failed to correctly locate the lesions in the descending colon (17/32 vs 26/32, *p* = 0.031).

However, the agreement between the two methods in comparison with the intraoperative findings demonstrated an almost perfect agreement between the two procedures (CT scan versus colonoscopy, weighted κ: 0.881).

The comparison between colonoscopy and computed tomography in accurate lesions localization is reported in Table 4.

**Table 4:** Comparison between colonoscopy lesion localization and CT scan

Localization (intraoperative)	Colonoscopy	CT scan	*p* value
Accurate localization	499	510	0.518*
Localization			
Caecum (118)	100	106	0.328
Ascending colon (144)	114	116	0.883
Hepatic flexure (42)	28	31	0.633
Transverse colon (65)	36	38	0.859
Splenic flexure (52)	29	29	1.000
Descending colon (32)	17	26	**0.031**
Sigma (161)	141	137	0.627
Recto-sigmoid junction (47)	34	27	0.194

*Analyses are performed on 661 patients (lesions seen at CT)

### Intraoperative data

Open surgery was performed in 180 patients (24.8%), while laparoscopy was performed in 529 (72.6%) and robotic-assisted surgery was performed in 19 (2.6%).

Among the cases in which colonoscopy or both methods incorrectly localized the lesions, a change in intraoperative management was necessary in 29 cases (4%). In fact, 4 minimally invasive procedures were converted to open surgery; in three cases, intraoperative colonoscopy was needed, and in the other 22 cases, the planned resection was modified.

Considering the real localization of the lesions, in the 4 converted cases the error differed by two colonic segments (ascending colon vs middle transverse colon); in the other remaining 25 cases the localization error differed by only one segment (right colon vs transverse and left colon vs recto-sigmoid junction/splenic flexure).

The intraoperative data are shown in Table 5.

**Table 5:** Changes in on-table management

Planned	Modified	Reason	No of cases
Right hemicolectomy	Extended right hemicolectomy	Lesion of the transverse	5
	Conversion to open	lesion of the transverse, technical difficulties	4
	Transverse colon resection	Lesion in middle transverse	1
Left hemicolectomy	Splenic flexure resection	Lesion in the splenic flexure	6
	Extended left hemicolectomy	Lesion in the transverse	5
	Intraoperative coloscopy	Lesion in the splenic flexure/transverse	2
	Anterior resection with AMI preserving	Lesion of the recto-sigmoid junction	5
	Intraoperative colonoscopy	Lesion of the sigma	1

*IMA* inferior mesenteric artery

### Multivariate analyses

Multivariate analyses showed that neither colonoscopy and CT were significantly influenced by any of the patients’ characteristics or pathological data. The results of the multivariate analysis are shown in Table 6.

**Table 6:** Multivariate analyses

Factors	Colonoscopy *p* value (OR; 95% CI)	CT scan *p* value (OR; 95% CI)
Age	0.59 (0.981; 0.962,1.001)	0.129 (0.985; 0.967, 1.004)
BMI	0.684 (1.010; 0.964, 1.058)	0.822 (0.995; 0.950, 1.041)
Gender	0.956 (1.011; 0.679, 1.505)	0.442 (1.165; 0.789, 1.719)
ASA score	0.931 (1.016; 0.716, 1.440)	0.080 (1.345; 0.965, 1.875)
Previous colonic resection	0.428 (1.557; 0.521,4.654)	0.702 (0.779; 0.216, 2.803)
Obstructing mass	0.359 (0.653; 0.263; 1.622)	NP
Bowel preparation	0.839 (0.978; 0.786, 1.216)	NP
Complete colonoscopy	0.069 (0.433; 0.176, 1.066)	NP
T stage	0.312 (0.892; 0.716, 1.113)	0.274 (1.140; 0.901, 1.442)
*D*_max_	0.859 (0.991; 0.899, 1.093)	0.549 (1.028; 0.938, 1.127)

*Dmax* maximum diameter of the lesion, *NP* not performed

## Discussion

To the best of our knowledge, this is the largest series to compare CT and conventional colonoscopy in lesion localization.

Correct preoperative lesion localization is one of the most important aspects for optimal preoperative surgical planning.

In fact, incorrect localization is a cause of on-table alterations in surgical management, especially in minimally invasive surgery, leading to the need for an additional trocar or a different type of robotic docking [9, 12].

Although colonoscopy is considered the gold standard in the detection of colorectal lesions, little is known about its accuracy in lesion localization [1].

Additionally, it has not been extensively clarified whether CT scans could be considered an aid to correctly localize colonic lesions [6, 9].

Several publications have demonstrated variability in the accuracy of colonoscopy with a range from 79 to 88% [3–5, 13–15] that decreases to 63.5% in the transverse colon [16].

In contrast, few studies have investigated the accuracy of CT in determining lesion location, reporting an overall accuracy ranging from 42.3 to 90.5% [6, 10, 16, 17].

However, in recent years, the accuracy of CT and colonoscopy in localizing lesions has been questioned [6, 10, 16–19].

Lee et al. [6], in a retrospective analysis of 104 patients affected by colon cancer, reported an accuracy of 79.8% for colonoscopy and 50% for CT, with missed lesions in 32.7% of cases.

Similarly, Feuerlein et al. [10] analysed data from 46 patients and demonstrated that conventional colonoscopy and CT imaging had an accuracy of 78.7% and 67.4% in localizing colonic lesions, respectively.

A lower accuracy rate was observed by Solon et al. [16] in their analysis of 101 patients with right colon cancer. In fact, the author reported an overall accuracy of 43% for CT and 59.5% for endoscopy.

In contrast, higher accuracy rates for colonoscopy and CT scans in localizing sigmoidal and rectal lesions was demonstrated by Loffeld et al. [17]. The author reported an overall accuracy of 87.5% for colonoscopy and 90.5% for imaging.

More recently, Johnstone et al. [18] demonstrated in a prospective multicentric analysis of 79 patients with colorectal cancer that colonoscopy accurately located 81% of tumours, while CT was unable to identify the primary tumour in 23.1% of cases, with an overall accuracy of 88.3% among cases in which the lesion was detected.

Finally, Moug et al. [19] analysed 364 patients with colorectal cancer in a large prospective study and demonstrated an overall accuracy of 82% for colonoscopy and 59% for CT. However, when considering only the lesions that could be seen on CT scans, the accuracy increased to 80%.

Considering our results, the accuracy of tumour localization is in the range reported in the current literature.

In fact, based on the data of 729 patients, colonoscopy and CT scans were accurate in 74.6% and 70.1% of cases, respectively. However, when considering only the lesions that were detected by CT, the accuracy of this imaging tool in localizing colonic lesions increased to 77.2%.

The comparison between CT and colonoscopy did not show a significant difference in terms of accuracy in localizing visible lesions, even when considering only the visible lesions and the patients who underwent complete colonoscopy. However, when considering the intraoperative localization results as the reference standard, the comparison between the two preoperative tools showed that colonoscopy significantly failed to correctly locate the lesions in the descending colon.

This result is in accordance with the current literature, which has demonstrated that colonoscopy has a lower accuracy in localizing lesions in the colonic segments far from anatomic landmarks [16].

By analysing the potential impact of risk factors on incorrect localization, we showed that none of the patients’ characteristics or pathologic data significantly influenced lesion localization with either preoperative tool. Our results are in contrast with the current literature, which has proposed several risk factors [3, 4, 8, 15].

Vaziri et al. [3], in their analysis of 374 patients, proposed increased age as a potential risk factor for incorrect lesion localization but found that patient sex did not significantly impact lesion localization.

In contrast, Piscatelli et al. [15] and Borda et al. [4] showed that age did not impact lesion localization, but they demonstrated that the significant influencing factors were previous abdominal surgery and incomplete colonoscopy, respectively.

Finally, Bryce et al. [8] noted incomplete colonoscopy as a unique risk factor for incorrect lesion localization.

To the best of our knowledge, this is the largest series to compare CT and conventional colonoscopy in lesion localization.

However, a major limitation of the study has to be addressed. Indeed, as a multicentric study, the different experiences of the involved specialists among the centres could represent an important concern.

In conclusion, we did not identify that CT has an advantage in localizing colonic tumours. In this setting, colonoscopy should be considered the standard reference to properly localize the lesions; however, to better localize lesions in the descending colon, CT could be considered a valuable tool to improve the accuracy of lesion localization.

## Data Availability

Data are available to the corresponding Author.
